# Anemia in Neonatal Piglets: Different Iron Supplementation Strategies on Growth and Hematological Parameters of Piglets

**DOI:** 10.3390/vetsci13020146

**Published:** 2026-02-03

**Authors:** Kobe Buyse, Geert P. J. Janssens, Ruben Decaluwé, Bart Pardon, Ioannis Arsenakis, Dominiek Maes

**Affiliations:** 1 Animal Science Unit, Institute for Agricultural, Fisheries and Food Research, Burg. Van Gansberghelaan, 9090 Merelbeke-Melle, Belgium; 2 Department of Veterinary and Biosciences, Faculty of Veterinary Medicine, Ghent University, Heidestraat 19, 9820 Merelbeke-Melle, Belgium; 3 Trouw Nutrition, Akkerhage 4, 9000 Ghent, Belgium; 4 Department of Large Animal Internal Medicine, Faculty of Veterinary Medicine, Ghent University, Salisburylaan 133, 9820 Merelbeke-Melle, Belgium; 5 Laboratory of Anatomy & Physiology of Farm Animals, Department of Animal Science, School of Animal Biosciences, Agricultural University of Athens, Iera Odos 75, 11855 Athens, Greece; 6 Unit of Porcine Health Management, Department of Reproduction, Obstetrics and Herd Health, Faculty of Veterinary Medicine, Ghent University, Salisburylaan 133, 9820 Merelbeke-Melle, Belgium

**Keywords:** piglets, iron supplementation, hematological parameters, performance

## Abstract

Piglets are highly susceptible to iron deficiency. This randomized clinical trial evaluated the effectiveness of four iron dosing schemes in preventing anemia. Two herds with different farrowing management systems were included. In each herd, 40 litters (6 piglets/litter) were selected on day 3 of age. A 2 × 2 factorial design was applied, combining two intramuscular iron dextran injection schemes [37.5 mg Fe/kg (low injection; LI) or 150 mg Fe/kg (high injection; HI)] with two oral ferrous sulphate feed supplementation schemes [125 mg Fe/kg (low feed; LF) or 200 mg Fe/kg (high feed; HF)]. Blood samples were collected at 4 and 20 days of age, and piglets were weighed at 3 and 20 days. Data were analyzed using linear mixed models, with significance set at *p* < 0.05. In Herd A, HI-LF piglets showed increased body weight, whereas no growth differences were observed in Herd B. Creep-feed intake did not differ between treatments. HI consistently improved red-cell indices in Herd A, while in Herd B LI piglets initially showed higher values at day 4, but HI piglets surpassed them by day 20. Leukocyte responses were limited. High-dose iron injections were effective in preventing anemia, while oral supplementation had minimal impact.

## Simple Summary

Young piglets are very prone to iron deficiency, which can lead to anemia and poor growth. Current practices include supplementing iron via injection. However, there is a risk of overdosing many piglets. This study tested an alternate method of giving iron to piglets by combining two methods. Piglets on two farms received iron either as an injection (low or high dose) added or not with supplemented iron via the creep feed. Their weight and blood values were checked during the first three weeks of life. The results showed that high-dose iron injections were the most effective at preventing anemia. Adding more iron to the feed has little effect on growth or blood health. Overall, the study shows that iron injections are more important than voluntary intake of iron-supplemented creep feed in protecting piglets against anemia.

## 1. Introduction

Its common practice in commercial pig herds to supplement neonatal piglets with iron via parenteral administration. Iron is an essential metallic element for the growth and well-being of suckling piglets. Piglets are born with very low iron body reserves. Additionally, colostrum and milk contain low levels of iron, and piglets in commercial herds cannot acquire iron from the soil as is the case in nature [1,2]. Therefore, intramuscular supplementation of iron is a defendable strategy, although purely oral supplementation seems possible.

Iron-deficient piglets cannot synthesize an adequate amount of hemoglobin (Hb) and have fewer red blood cells (RBCs) than normal. This condition is described as iron-deficiency anemia (IDA) or otherwise termed as hypochromic microcytic anemia [2,3,4,5]. Clinical signs may be visible from 10–14 days onwards with pale mucosa and, in later stages, pale skin as the main signs [1,2,3,6]. Severely affected animals can become lethargic and show high heart rate and dyspnea, resulting in poor performance. Upon necropsy, anemic pigs may show hypertrophy of the heart, anasarca, watery blood, and general signs of hypoxia [2,3]. The lower iron reserve could result in a lower immune response and predispose to secondary illness such as diarrhea.

The ideal supplemented iron dose depends on age, daily gain, administration route, and bioavailability of the iron source. Different products such as iron dextran, iron fumarate, iron glutamate, and gleptoferron are used [2,7]. A dosage of 200 mg/animal is often recommended as it results in optimal performance and hematology parameters [1,2,8]. However, a parenteral bolus of 200 mg could cause toxicity in 90% of the supplemented piglets, with possible liver damage due to oxidative stress and risking the formation of abscesses, nerve damage and necrosis [9,10,11]. Other disadvantages of parenteral iron supplementation include the iatrogenic spread of pathogens such as *Mycoplasma suis* between piglets, the extra labor, and the effect that a single large iron dose could inhibit the uptake of intestinal iron due to increased hepcidin levels [12].

When body iron levels increase, a hormone called hepcidin is produced which blocks expression of iron receptors in the gastro-intestinal tract, prohibiting enteral iron absorption via milk or feed. This negative feedback mechanism is needed to prevent iron intoxication. Nonetheless, Starzynski et al. [13] showed that injection of 150 mg iron-dextran/kg body weight at 3 days of age induces high production of hepcidin up until weaning and beyond. By supplementing only 25% of that dose, hepcidin levels remained low or below detection, even when a second injection of a 25% dose was given at 14 days of age. Piglets subjected to the low supplemented iron group showed similar to improved hematological results at 21 and 28 days of age. This indicates that the high intramuscular supplementation in the neonatal period might not be the best way to control iron storage in neonatal piglets. The study of Starzynski et al. [13] has, however, not investigated a common iron supplementation protocol applied in commercial herds: the application of an intramuscular iron injection at 3 days of age in combination with the provision of prestarter creep feed supplemented with iron from the third day of age till weaning.

Thus, the aim of this study was to investigate whether we can optimize current iron supplementation management in pre-weaned piglets to prevent subclinical anemia and reduced performance by combining different levels of intramuscular and oral iron supplementation. Body weight and Hb concentration were taken as main parameters to assess performance and anemia status, respectively.

## 2. Materials and Methods

### 2.1. Herd Description

This study was conducted in two commercial herds comprising 400 (Herd A) and 1750 (Herd B) sows. This inclusion of two farms was to assess the potential different outcomes between management practices. Both herds were managed by all-in/all-out, operated a 2-week batch production system and weaned at 21 days of age. A description of both herds together with the piglet management practices applied from birth to the nursery period are presented in Table 1.

Both herds were included in the study on the basis that neither of them faced recent outbreaks with viral pathogens, such as porcine reproductive and respiratory syndrome virus (PRRSV), porcine circovirus type 2 (PCV-2) and porcine epidemic diarrhea virus (PEDV). Additionally, in the farrowing units of both herds there were no other disease manifestations observed in the sows (e.g., hypogalactia or lameness) or the piglets (e.g., diarrhea, vomiting, joint swelling, or skin lesions). Both herds did not use any farrowing induction protocols in the sows and were highly productive, with at least 28 piglets weaned per sow per year. Cross-fostering was not applied to any litter included in the study.

### 2.2. Study Population and Experimental Design

This study was designed as a randomized clinical trial, conducted and reported in accordance with established reporting guidelines for randomized controlled trials. Within each herd, 40 sows that belonged to parities between one and seven were selected to participate in the study. The sows met the following inclusion criteria: no farrowing induction, farrowing on the same day, gestation length of 114–116 days, maximum farrowing duration of 6 h, a minimum of 12 liveborn and a maximum of 3 stillborn piglets per litter and a sow body condition score ranging between 2 and 4 according to Maes et al. [14].

The selected farrowing crates were randomly divided into four different groups (n = 10; 60 piglets per treatment). More specifically, a 2 × 2 factorial design was used; two different intramuscular iron dextran injection schemes [37.5; (LI) or 150; (HI) mg Fe-dextran/kg] were combined with two oral ferrous sulphate schemes [125; (LF) or 200 (HF) mg Fe-sulphate/kg, as creep feed; Table 2]. Injections with iron dextran (Ferraject 200^®^, Dechra, Northwich, United Kingdomwere applied in both herds at the third day of age. They were injected intramuscularly in the neck region behind the right ear (BD Microlance 20 G 25 mm, BD, Eysins, Vaud, Switzerland) The creep feed (Trouw Nutrition, The Netherlands) was provided ad libitum from the third day of age until the day of weaning in a trough plate at the corner of each farrowing crate. The different treatment groups were formed as follows: LI-LF, LI-HF, HI-HF and HI-LF.

Within each litter 6 piglets were selected based on weight and gender, resulting in a total of 240 piglets (60 piglets per treatment). All piglets of a litter were weighed and, subsequently, six piglets were selected to get a representative sample within the litter: two small (lower quartile of the litter), two medium (median), and two large (upper quartile of the litter) piglets. If possible three male and three female piglets were selected. Piglets with diarrhea, arthritis, congenital disorders, and runt piglets were excluded. This selection ensured a representable sample of the litter; however, these factors were not included as fixed effects in the model. The six piglets remained with their sows from farrowing until weaning and received the same treatment. The other piglets were not included in the study. They stayed in the farrowing pen and received the conventional iron treatment (1 mL of iron dextran per piglet), although they could eat from the experimental creep feed.

In each herd, the design of all farrowing crates and pens in which the sows and piglets were housed was identical for all four treatment groups. All piglets included in the study were ear-tagged on the third day of age so that they could be identified during the suckling period. The study was approved by the ethical committee for animal experiments of the Faculty of Veterinary Medicine, Ghent University (EC2016/100).

### 2.3. Sample and Data Collection

After supplementing the treatments on day 3, weighing and sampling were performed in a blinded manner with respect to the treatment by the first author and animal caretakers during the experiment. Blinded ear tag numbers of each piglet were used throughout the study (data collection and sample analysis). Individual body weight was measured at 3 and 20 days of age to determine the average daily weight gain (ADG; g/pig/day) of each treatment group.

Records of mortality rates were kept during the whole duration of the study, from the third until the twentieth day of age. The ear tag of each animal was noted, together with the date of death. The average daily creep feed consumption per piglet was estimated based on the amount of feed consumed per litter from the third until the twentieth day of age divided by the litter size at day 20 and number of days in trial.

On the 4th day and the 20th day of age, whole blood samples were taken from the *vena jugularis* (BD Microlance 18 G 40 mm, BD, Eysins, Vaud, Switzerland) with an EDTA-tube (BD Vacutainer K2E (EDTA) 8 mL, BD, Eysins, Vaud, Switzerland). Samples were refrigerated (4 °C) for 12 to 24 h until the analysis. Tubes were placed on a roller mixer until samples were homogenized. Full hematology was performed on the individual blood samples with a benchtop analyzer (IDEXX ProCyte Dx, IDEXX Laboratories, Westbrook, ME) at the faculty of veterinary medicine (Ghent University). With Hb concentration (g/dL) as the main parameter, the following additional parameters were analyzed: hematocrit (Hct, %), red blood cell count (RBC, M/µL), reticulocyte count (RET count, K/µL), reticulocyte percentage (RET %, %), mean corpuscular hemoglobin (MCH, pg), mean corpuscular hemoglobin concentration (MCHC, g/dL), mean corpuscular volume (MCV, fL), white blood cell count (WBC, K/µL), lymphocyte count (LYMPH, K/µL), neutrophile count (NEUT, K/µL), and monocyte count (MONO, K/µL). The threshold for anemia was set at 8 g Hb/dL [15].

### 2.4. Statistical Analysis

Data was analyzed with R studio for Windows (2 July 2022). The two herds were analyzed separately. The farrowing crate identified with sow number was the experimental unit. Piglets were nested per sow as a random effect and initial body weight was used as a covariable for the hematology parameters and body weight at the end of the trial. Body weight and Hb were the main parameters investigated to assess anemia and health of the piglet. Data were analyzed using the interaction of injection dose and feed content as fixed effects. Data were analyzed with least-squared linear mixed model regression. The percentage of piglets with anemia (Hb < 8 g/dL) and the mortality were analyzed using cumulative logistic regression with the same fixed effects. When significant, variance analysis with Tukey post-hoc correction was used for post hoc pairwise comparison. Age was not included in the model and analyzed as separate datasets. All data were checked for outliers and visually checked for a normal distribution of the residuals with distribution and Q-Q plots. Pearson correlation coefficients were calculated between hemoglobin concentration and leucocyte count. Differences were considered statistically significant at *p* < 0.05.

## 3. Results

### 3.1. Performance

In Herd A, the HI groups had significantly larger body weights at day 20 compared to the LI groups (*p* = 0.032) (Table 3). Concerning the ADG, in Herd A, groups LI-HF and LI-LF performed significantly lower compared to both the groups receiving HI (*p* = 0.006). There was a trend towards higher ADG in the LF groups (*p* = 0.068). This could be attributed to the fact that these groups started with a lower average weight at day 3 (*p* = 0.003). In Herd B there were no significant differences in body weight of the selected piglets among groups at day 3 and day 20 (Table 3), and there were no significant differences in the ADG between groups (Table 3). In both herds, creep feed consumption was unaffected by the treatments. A larger daily creep feed consumption in Herd B was noted compared to Herd A. Only in Herd A, high-iron injection reduced pre-weaning mortality (*p* = 0.045).

### 3.2. Hematology

Based on hematology data both herds reacted differently to the treatments (Table 4). In Herd A, piglets receiving HI had overall higher Hb (*p* < 0.001), Hct (*p* < 0.001), RBC (*p* < 0.001) and Ret (*p* < 0.001) on both ages, day 4 and day 20, except for Hb on day 4 where no significant differences between treatments were noted. Neither iron dose in feed nor its interaction with injection dose affected any of the iron status parameters in Herd A. This was similar in Herd B, apart from lower Hb (*p* = 0.011), Hct (*p* = 0.001) and RBC (*p* = 0.069) on day 4 due to high-iron injection, whereas on day 20 these parameters were significantly higher in the HI groups similar to Herd A. Ret showed less responsiveness to the treatments with only a higher Ret count on day 20 in the HI groups (*p* = 0.012).

**Figure 1:**
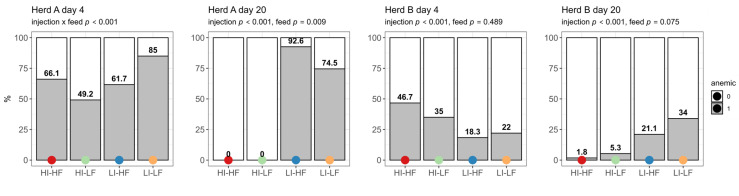
Effect of injection (HI: 150 mg/kg BW, LI: 37.5 mg/kg BW) and its interaction with feed treatments (HF: 200 mg/kg, LF: 125 mg/kg) on prevalence of piglets with anemia (score 1) on the first day after injection (day 4) and before weaning (day 20) (n = 60).

The prevalence of anemic piglets based on Hb concentration was high on day 4 in Herd A and showed interaction between treatments, with LI-LF having the highest prevalence (*p* < 0.001) (Figure 1). The prevalence of anemia increased for the LI treatments in this herd on day 20 (*p* < 0.001) and no piglets showed anemia in the HI groups. LF as a second main effect reduced the prevalence (*p* = 0.009). The prevalence of anemia was significantly higher for the HI groups in Herd B on day 4 (*p* < 0.001). However, on day 20 it was the LI groups that showed the highest prevalence (*p* < 0.001).

The effect of iron dose was also noted on the size and Hb content of red blood cells (Table 5). MCV was not significantly different on day 4 in Herd A; however, it was larger at day 20 in the HI groups (*p* < 0.001). Nevertheless, on day 4 LI groups (*p* < 0.001) showed significantly higher Hb content. In contrast, on day 20, HI groups showed the highest MCH (*p* < 0.001). The combination of these parameters resulted in a higher MCHC in piglets of the LI groups (*p* < 0.001) and an interaction between injection and feed (*p* < 0.001) on day 20, with HI-HF having the highest MCHC followed by, in decreasing order, LI-LF, HI-LF and LI-LF. Piglets of Herd B reacted somewhat similar, with day 20 HI groups showing the highest MCV (*p* < 0.001), MCH (*p* < 0.001) and MCHC (*p* < 0.001). However, on day 4, MCV was highest in LI groups (*p* = 0.004) and treatments tended to interact for MCH (*p* = 0.034; however, post hoc tests were not significant). Similarly to the other hematology parameters, no effects of feed dosage were observed.

Lastly, different white blood cell counts were recorded (Table 6). In Herd A no significant effects of the treatments were noted on most of the parameters except on day 20, where monocytes (*p* = 0.003) were higher in the LI groups. In contrast, there was a distinct feed effect in Herd B. HF groups had larger leucocytes (*p* = 0.041), neutrophils (*p* = 0.031) and monocytes (*p* < 0.001) counts on day 4. On day 20 no significant effects were observed except for neutrophils that were higher in the HI groups (*p* = 0.042). Based on all data there was a correlation between hemoglobin as marker for anemia and leukocytes (r = 0.118, *p* < 0.001), lymphocytes (r = 0.288, *p* < 0.001), neutrophils (r = −0.149, *p* < 0.001), eosinophils (r = 0.179, *p* < 0.001) and monocytes (r = 0.021, *p* = 0.532).

## 4. Discussion

Under the conditions of the present study, iron injection was clearly more efficient than dietary iron supplementation, and the high iron injection dose seemed necessary to overcome anemia by the end of the suckling phase based on hemoglobin concentration. Despite the similar experimental setup and selection protocol, some facets of the effects of iron supplementation treatment differed between the two herds, pointing to residual variation due to a plethora of uncontrolled factors such as housing conditions, sow genotype, farrowing management and weight of the neonatal piglets.

**Figure 2:**
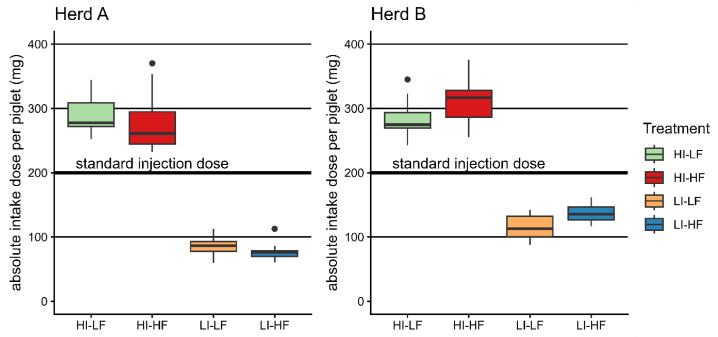
Boxplots of the absolute applied total doses per piglet (injection and mean litter feed intake) compared with the standard injection dose of 200 mg/animal.

In Herd A, piglets with low injection dose showed lower body weight and ADG and higher mortality at weaning. This effect was absent in Herd B. The feed effect on body weight seen in Herd A may be due to an imbalance of body weight despite random selection of the litters on day 3. Overall, independent of treatments, piglets consumed more iron-enriched feed in Herd B than Herd A, implying a higher absolute dietary total iron intake above the parenteral treatment demonstrated in Herd B by HI-HF and LI-HF compared to the LF groups (Figure 2). This effect was negligible in Herd A. Nevertheless, apart from the performance results from both herds, it was shown that injection of a high iron dose caused a better development of the hematology parameters at weaning age. In Europe, piglets typically receive a standard dose of 1 mL with 200 mg of iron as iron dextran independent of bodyweight. On an absolute basis, this dose would represent the mean of the treatments used in this trial (Figure 2). Based on the absolute amount received and the method of supplementing, we could conclude that the dose yielded by voluntary dietary intake is only secondary to parenteral administration.

The addition of an iron-enriched creep feed to the piglets yielded little added effect on the lower parenteral doses even though lower parenteral dosages might reduce hepcidin, thereby omitting the negative feedback loop of intestinal iron uptake [13]. Moreover, the overall main effects of dietary iron intake on performance and hematology were limited, which can be explained by two mechanisms.

Firstly, the lower expression of the selective divalent metal transporter (DMT-1) until the age of 26 days reduces the absorption of inorganic iron [16]. Iron components ingested before gut closure at 24 h after birth are non-selectively absorbed with pinocytosis in the duodenum [17,18,19]. In the ileum, iron could be absorbed non-selectively up to three weeks of age when piglets were in good health [17]. Effects of dietary iron would only be visible after 2 to 3 weeks, which would barely exceed the length of this study [8]. The restricted intestinal uptake of iron could be ameliorated by using amino-acid-chelated iron [20,21,22] or iron bound to lactoferrin [23,24]. In addition, mediating a lower stomach pH by dietary intervention would also improve iron availability [12].

Secondly, feed intake of creep feed is typically low and highly variable at the start of the lactation period as piglets receive most of their nutrients from the sow [25,26]. This makes the uptake of iron via dietary supplementation more prone to variation [11].

The strong iron barrier in the enterocytes and the variable feed intake might be the cause of the lack of impact on performance and other parameters. The creep feed was presented in this study on voluntary basis from day 3 onwards, potentially missing the window of efficient enteral uptake of important mineral complexes hours after birth, leading towards an absence of effect on performance [27]. The more accurate force-fed iron supplements, in contrast to voluntary uptake, may show opportunities to reduce the use of parenteral injection [27,28].

The effect of injection dose on hematology was highly noticeable in both herds. A parenteral dose of iron dextran would cause a higher production of red blood cells, as proven by the higher red blood cell count, hematocrit and reticulocyte count. In Herd A, at both timepoints, these parameters were positively affected in the HI groups, whilst, in Herd B on day 4, LI showed a higher concentration of Hb, Hct and RBC. Nevertheless, based on the Hb on D4, piglets in Herd A could already be classified as anemic regardless of treatment; this was absent in Herd B. Differences between herds in the presence of anemia were also evident at D20 with the LI doses. In Herd B, no piglets were anemic, whereas, in Herd A, HI mitigated the effects of lower iron availability, while LI piglets remained anemic. Therefore, presence of anemia not only depended on the supplemented iron but also on the natural iron reserve of neonatal piglets. The source of the discrepancy between two herds on day 4 is unclear but may be related to initial iron reserves marked by ferritin or management practices such as the presence of chirurgical castration with the additional blood loss and the parenteral antibiotic administrations in Herd A, which may induce an acute response. This was also reflected in the red blood cell physiology of Herd A with smaller red blood cells on day 4. The RBCs contained less hemoglobin in the high injection group due to the higher production rate. In contrast, at day 20, the RBCs were larger, with a higher concentration of hemoglobin inside the cells, likely because, at that time, the iron injection was fully absorbed and incorporated. We postulate that the injection of high iron dose on day 3 stimulated erythropoiesis on day 4, as indicated by high reticulocyte count and production of a larger number of smaller less matured red blood cells indicated by smaller MCV, MCH and MCHC. In Herd B, there was no chirurgical castration and therefore no iron loss due to blood loss and healing processes. Therefore, in the HI groups, an acute negative feedback towards erythropoiesis might have taken place. This was reflected by the absence of significant effects on reticulocyte count on day 4. Despite the differences in acute response on red blood cell count, the same feedback response on MCV, MCH and MCHC was noted in Herd B on day 20. The high iron supply of the HI dose would keep on stimulating erythropoiesis and therefore increasing Hb and Hct with larger cells and increased MCHC by the end of the suckling period. With this assumption the size and Hb concentration would thus be useful in later stages to assess iron status in piglets [4]. Because of the lower Hb concentration and the smaller size of the RBCs, this anemia could be categorized as hypochromic and microcytic. Based on the management differences between herds such as castration, additional parenteral injections would be mandatory in discussing iron supplement strategies.

Blood parameters were presented in absolute and not relative numbers to avoid influence from other blood cells [4]. From the investigated parameters, hematocrit was the most sensitive method for diagnosing anemia in the short term because of its rapid decrease [8]. The Hb concentration decreases slower and is therefore visible only in later stages [1,3]. This was confirmed in this study, as Hb concentration only decreased on day 20, especially in Herd A. Hematocrit values, however, are dependent on the hydration status of the piglet and, in this sense, Hb is considered a better parameter to assess anemia. A threshold of 8 g/dL Hb has been proposed as an indication of anemia in piglets [15]. At the first sampling, piglets showed a high prevalence of anemia, and this prevalence remained high at later stages in piglets with low-dose injections. However, supplementing iron in the diet might reduce the prevalence of anemia at weaning age. Absolute reticulocytes would also be a good parameter to measure anemia. They represent the degree of erythropoiesis, especially in the first week at the peak of red blood cell production [4], and are only a little influenced by other parameters [29]. A lower number of reticulocytes, as found in this study, points to a non-regenerative anemia [4,5].

The white blood cells of Herd A were little affected; however, in the LI group, higher monocyte counts were found, suggesting a chronic inflammation status. The mortality rate was also higher in the LI group of Herd A. The cell numbers were considerably higher than in Herd B, where mortality remained low. In Herd B, added iron in the feed as the main effect caused significant increases in white blood cell counts, neutrophils and monocytes. One possible reason is the potentially higher challenge of microbial content because of the higher iron amount in the intestinal tract as iron is a nutrient source for many bacteria. This would reflect a higher non-specific immunity response as the lymphocyte count did not change with the various doses of iron supplementation. However, one study suggested that iron supplementation may improve intestinal health with increased villus length means of mucosal morphology [30].

In Herd B, monocyte count tended to be increased by the high injection dose, proving the adverse effect of the injection of a foreign body. In contrast with other studies [29], no associations between anemia, marked by the correlation between hemoglobin concentration and immune response, could be found. Possibly, the effects on white blood cells are variable and dependent on many other factors such as immunological challenges [3].

## 5. Conclusions

This study highlights the significant role of intramuscular iron supplementation in preventing anemia in neonatal piglets, yet with marked differences in efficacy between herds. Providing iron by injection to the piglets was more effective and caused a better hematological response than supplementing dietary iron, possibly due to factors such as limited voluntary intake and regulatory responses upon intestinal iron absorption. Nevertheless, performance until weaning was not different between the treatment strategies in one of the herds and therefore further research is warranted to assess strategies of lowering the parenteral dose and promoting dietary iron intake across herds.

## Figures and Tables

**Table 1:** Herd description and piglet management practices.

	Herd A	Herd B
Number of sows	400	1.750
Breed of sows	Danbred, Topigs 20	PIC
Breed of boars (sperm)	Belgian Piétrain	Belgian Piétrain
Production system in the sows *	2 weeks	2 weeks
Management of the piglets		
Tail docking	day 2–4	day 2–4
castration	Chirurgical (day 2–4)	Immunocastrated with Improvac (week 10 and 18)
Antimicrobial use	Neomycin sulfate—procaine benzylpenicillin (IM) (Neopen^®^, Intervet Int)	no use of antimicrobials
Anticoccidial use		toltrazuril (Dozuril, Dopharma)
Vaccinations day 14		
M hyo, PCV-2 and PRRS(V) **	Ingelvac FLEXcombo (Boehringer), Ingelvac MLV (Boehringer)	Ingelvac 3FLEX (Boehringer)

* In a two-week batch production system, groups of sows farrow every two weeks. ** M hyo, Mycoplasma hyopneumoniae; PCV-2, porcine circovirus type 2; PRRS(V), porcine reproductive and respiratory syndrome virus.

**Table 2:** Composition of the experimental creep feed (fresh matter base) for the suckling piglets in herd A and B.

Dry matter (g/kg)	925
Crude protein (g/kg)	190
Crude Fat (g/kg)	100
Crude Ash (g/kg)	55
Crude fibre (g/kg)	20
Iron sulfate (mg/kg)	125 (LF)–200 (HF) ^§^

^§^ LF: low-dosed feed; HF: high-dosed feed.

**Table 3:** Effect of the interaction between injection dose (High, HI: 150 mg/kg BW, Low, LI: 37.5 mg/kg BW) and iron content in the feed (High, HF: 200 mg/kg, Low, LF: 125 mg/kg) on mean body weight (BW, kg) of piglets, average daily gain (ADG, g/piglet/day), total creep feed intake (TCFI, g/piglet/day) and mortality of the selected piglets per treatment (%) during the first three weeks in the farrowing crates (D3-20) (n = 10; 60 piglets).

	HI		LI			*p*-Value ^1^		
Herd A	HF	LF	HF	LF	SEM	IxF	I	F
BW D 3	1.63 ^b^	1.86 ^a^	1.59 ^b^	1.81 ^a^	0.06	0.180	0.545	0.003
BW D20	5.70 ^A^	5.75 ^A^	5.29 ^B^	5.34 ^B^	0.17	0.381	0.032	0.787
ADG	215 ^A^	233 ^A^	188 ^B^	206 ^B^	9	0.757	0.006	0.068
TCFI *	6.97	7.89	5.90	6.83	0.72	0.586	0.210	0.274
mortality	0.23 ^B^	0.56 ^B^	1.67 ^A^	1.39 ^A^	0.07	0.521	0.045	1.000
Herd B								
BW D3	1.53	1.54	1.46	1.47	0.06	0.816	0.260	0.871
BW D20	5.42	5.14	5.57	5.29	0.17	0.372	0.440	0.143
ADG	221	204	223	206	39	0.277	0.812	0.102
TCFI *	21.9	24.2	23.7	26.1	1.3	0.183	0.228	0.131
mortality	0.63	1.16	1.16	2.11	0.90	0.142	0.223	0.223

^1^ I: injection; F: feed; IxF: interaction. Different lowercase superscripts indicate significant differences among feed levels (*p* < 0.05). Different uppercase superscripts indicate significant differences among injection treatments (*p* < 0.05). * of the total litter.

**Table 4:** Effect of interaction between injection dose (High, HI: 150 mg/kg BW; Low, LI: 37.5 mg/kg BW) and iron content in the feed (High, HF: 200 mg/kg; Low, LF: 125 mg/kg) on hemoglobin (Hb, g/dL), hematocrit (Hct, %), red blood cell count (RBC, M/µL) and reticulocyte count (Ret, K/µL) of piglets on the first day after injection (day 4) and before weaning (day 20) (n = 60).

		HI		LI			*p*-Value ^1^		
Herd A	Day	HF	LF	HF	LF	SEM	IxF	I	F
Hb	D4	7.43	7.57	7.02	7.15	0.22	0.773	0.102	0.595
	D20	10.91 ^A^	11.21 ^A^	6.24 ^B^	6.54 ^B^	0.21	0.826	<0.001	0.214
Hct	D4	25.3 ^A^	24.5 ^A^	19.0 ^B^	18.2 ^B^	0.9	0.167	<0.001	0.491
	D20	33.9 ^A^	35.0 ^A^	20.4 ^B^	21.5 ^B^	0.7	0.055	<0.001	0.190
RBC	D4	3.28 ^A^	3.12 ^A^	2.39 ^B^	2.23 ^B^	0.10	0.086	<0.001	0.187
	D20	4.56 ^A^	4.64 ^A^	3.49 ^B^	3.56 ^B^	0.07	0.491	<0.001	0.389
Ret	D4	370 ^A^	358 ^A^	272 ^B^	260 ^B^	22	0.534	<0.001	0.624
	D20	307 ^A^	304 ^A^	118 ^B^	115 ^B^	15	0.615	<0.001	0.902
Herd B									
Hb	D4	8.10 ^B^	8.28 ^B^	8.70 ^A^	8.88 ^A^	0.20	0.324	0.011	0.453
	D20	11.46 ^A^	11.39 ^A^	8.98 ^B^	8.81 ^B^	0.27	0.355	<0.001	0.824
Hct	D4	23.8 ^B^	24.5 ^B^	26.5 ^A^	27.2 ^A^	0.7	0.633	0.001	0.365
	D20	37.8 ^A^	37.0 ^A^	31.4 ^B^	30.7 ^B^	1.0	0.747	<0.001	0.488
RBC	D4	3.21	3.29	3.38	3.46	0.08	0.983	0.069	0.421
	D20	5.08 ^A^	5.01 ^A^	4.73 ^B^	4.65 ^B^	0.09	0.918	0.001	0.490
Ret	D4	303	296	298	291	19	0.148	0.819	0.732
	D20	292 ^A^	297 ^A^	243 ^B^	249 ^B^	17	0.969	0.012	0.793

^1^ I: injection; F: feed; IxF: interaction. Different uppercase superscripts indicate significant differences among injection treatments (*p* < 0.05).

**Table 5:** Effect of interaction between injection dose (High, HI: 150 mg/kg BW; Low, LI: 37.5 mg/kg BW) and iron content in the feed (High, HF: 200 mg/kg; Low, LF: 125 mg/kg) on mean corpuscular volume (MCV, fL), mean corpuscular hemoglobin (MCH, pg) and mean corpuscular hemoglobin concentration (MCHC, g/dL) of piglets on the first day after injection (day 4) and before weaning (day 20) (n = 60).

		HI		LI			*p*-Value ^1^		
Herd A	Day	HF	LF	HF	LF	SEM	IxF	I	F
MCV	D4	77.3	79.6	79.9	82.2	1.6	0.791	0.164	0.217
	D20	74.6 ^A^	75.6 ^A^	58.7 ^B^	59.7 ^B^	1.4	0.094	<0.001	0.528
MCH	D4	22.7 ^B^	24.2 ^B^	29.7 ^A^	31.3 ^A^	0.7	0.420	<0.001	0.067
	D20	23.9 ^A^	24.3 ^A^	17.9 ^B^	18.3 ^B^	0.3	0.185	<0.001	0.352
MCHC	D4	29.6 ^B^	30.6 ^B^	37.4 ^A^	38.4 ^A^	0.9	0.315	<0.001	0.364
	D20	33.1 ^a^	31.3 ^b^	29.3 ^c^	31.9 ^ab^	0.4	<0.001	<0.001	0.006
Herd B									
MCV	D4	74.3 ^B^	74.8 ^B^	78.6 ^A^	79.1 ^A^	1.3	0.380	0.004	0.735
	D20	74.5 ^A^	73.9 ^A^	66.2 ^B^	65.6 ^B^	1.3	0.611	<0.001	0.710
MCH	D4	24.9	25.7	26.2	25.4	0.4	0.034 *	0.013	0.158
	D20	22.6 ^A^	22.8 ^A^	18.9 ^B^	19.1 ^B^	0.3	0.095	<0.001	0.667
MCHC	D4	34.3	33.9	33.0	32.6	0.6	0.612	0.053	0.530
	D20	30.4 ^A^	30.9 ^A^	28.6 ^B^	29.1 ^B^	0.4	0.184	<0.001	0.274

^1^ I: injection; F: feed; IxF: interaction. Different uppercase superscripts indicate significant differences among injection treatments (*p* < 0.05). * post hoc tests were not significant.

**Table 6:** Effect of interaction between injection dose (High, HI: 150 mg/kg BW; Low, LI: 37.5 mg/kg BW) and iron content in the feed (High, HF: 200 mg/kg; Low, LF: 125 mg/kg) on total white blood cell count (WBC, K/µL), lymphocyte count (Lymph, K/µL), neutrophil count (Neut, K/µL) and monocyte count (Mono, K/µL) of piglets on the first day after injection (day 4) and before weaning (day 20) (n = 60).

		HI		LI			*p*-Value ^1^		
Herd A	Day	HF	LF	HF	LF	SEM	IxF	I	F
WBC	D4	12.8	12.7	12.6	12.6	0.55	0.539	0.824	0.936
	D20	11.5	11.6	12.0	12.0	0.46	0.981	0.321	0.886
Lymph	D4	4.13	4.14	4.27	4.27	0.22	0.901	0.592	0.991
	D20	6.20	5.91	6.14	5.85	0.18	0.200	0.771	0.173
Neut	D4	8.22	8.25	7.92	7.99	0.38	0.962	0.496	0.886
	D20	4.87	5.25	5.33	5.71	0.36	0.506	0.257	0.360
Eo	D4	0.07 ^B^	0.09 ^B^	0.11 ^A^	0.13 ^A^	0.02	0.104	0.034	0.273
	D20	0.11	0.14	0.11	0.14	0.01	0.767	0.924	0.153
Mono	D4	0.53	0.53	0.47	0.46	0.04	0.959	0.120	0.849
	D20	0.33 ^B^	0.29 ^B^	0.43 ^A^	0.39 ^A^	0.03	0.740	0.003	0.219
Herd B									
WBC	D4	11.5 ^a^	10.1 ^b^	12.5 ^a^	11.1 ^b^	0.55	0.943	0.164	0.041
	D20	12.2	11.6	12.3	11.7	0.55	0.607	0.829	0.344
Lymph	D4	3.73	3.60	4.14	4.01	0.23	0.445	0.122	0.628
	D20	6.70	6.18	7.38	6.86	0.33	0.358	0.077	0.170
Neut	D4	7.09 ^a^	6.02 ^b^	7.66 ^a^	6.58 ^b^	0.43	0.809	0.256	0.031
	D20	4.91 ^A^	4.76 ^A^	4.25 ^B^	4.10 ^B^	0.25	0.857	0.042	0.628
Eo	D4	0.07	0.08	0.10	0.11	0.01	0.731	0.055	0.455
	D20	0.15	0.17	0.12	0.13	0.02	0.444	0.199	0.537
Mono	D4	0.61 ^a^	0.44 ^b^	0.54 ^a^	0.37 ^b^	0.03	0.294	0.071	<0.001
	D20	0.60	0.52	0.58	0.50	0.05	0.791	0.701	0.164

^1^ I: injection; F: feed; IxF: interaction. Different lowercase superscripts indicate significant differences among feed levels (*p* < 0.05). Different uppercase superscripts indicate significant differences among injection treatments (*p* < 0.05).

## Data Availability

The original contributions presented in this study are included in the article. Further inquiries can be directed to the corresponding author.
